# Trigemino-autonomic activation in a human trigeminal pain model

**DOI:** 10.1186/s12883-025-04147-y

**Published:** 2025-04-05

**Authors:** Stefan Evers, Achim Frese, Patrick Hornberg, Oliver Summ

**Affiliations:** 1 Faculty of Medicine, University of Münster, Münster, Germany; 2 Department of Neurology, Lindenbrunn Hospital, Lindenbrunn 1, 31863 Coppenbrügge, Germany; 3 Akademie für Manuelle Medizin, Münster, Germany; 4 Private Praxis, Emsdetten, Germany; 5 Department of Neurology and Research Center of Neurosensory Science, Carl von Ossietzky University Oldenburg, Oldenburg, Germany

**Keywords:** Capsaicin, Trigemino-autonomic symptoms, Cluster headache

## Abstract

**Background:**

Autonomic symptoms are mandatory for making the diagnosis of a trigemino-autonomic cephalalgia (TAC). These symptoms can occasionally also occur in migraine and facial pain disorders. This leads to the question whether the trigeminal pain itself can induce autonomic symptoms also in healthy subjects.

**Methods:**

We enrolled healthy subjects without a history of migraine or a TAC and provoked severe trigeminal pain by injection of 0.05 ml capsaicin (0.01%) into the right forehead. Autonomic symptoms occurring at the right eye or right nostril were registered until they disappeared. We also calculated an autonomic score for the frequency and duration of autonomic symptoms in an individual.

**Results:**

We enrolled 60 healthy volunteers (30 male, 30 female; mean age 28 +/- 5 years). All but two subjects developed at least one autonomic symptom after injection of capsaicin. One minute after injection, the pain was rated as 9.2 +/- 1.1 and 8.5 +/- 1.2 (scale from 0 to 10) in female and male subjects, respectively. The autonomic score was 4.4 +/- 1.6 and 1.7 +/- 0.9 for female and male subjects, respectively. All differences between female and male subjects were significant. Pain rating and autonomic score showed a significant positive correlation which remained significant even after adjusting for sex.

**Conclusions:**

Severe trigeminal pain was accompanied by autonomic symptoms in almost all subjects in this experiment. The pain rating and the severity of autonomic symptoms were significantly higher in female subjects than in male. The higher the pain the more severe was this autonomic activation. We conclude that activation of autonomic symptoms is an unspecific consequence of severe trigeminal pain. This does, however, not exclude the possibility that primary headache disorders might have an independent anatomic pathway to induce autonomic symptoms because these symptoms can, although very rarely, also occur without pain.

## Background

Ipsilateral and simultaneous autonomic activation to unilateral trigeminal pain is a typical feature of a group of primary headache disorders called trigemino-autonomic cephalalgias (TACs) [1]. The most common of these disorders is cluster headache. According to the International Classification of headache Disorders (ICHD-3) [2], the autonomic symptoms of TACs during the extreme pain attack comprise:

- conjunctival injection and/or lacrimation.
- nasal congestion and/or rhinorrhoea.
- eyelid oedema.
- forehead and facial sweating.
- miosis and/or ptosis.
- a sense of restlessness or agitation.

These symptoms indicate parasympathetic hyperactivity. In fact, the autonomic symptoms are so prominent that these headache disorders have been grouped within one chapter of ICHD-3. However, it is sometimes overlooked that other primary headache and facial pain disorders may exhibit autonomic symptoms as well [3]. Autonomic activation during severe unilateral migraine attacks [4]; [5] and first division trigeminal neuralgia [6] have also been described.

We were interested whether the occurrence of autonomic symptoms is restricted to primary headache disorders or whether also experimental pain in healthy subjects can induce such parasympathetic activation. Therefore, we designed a study on autonomic symptoms induced by capsaicin injected subcutaneously into the forehead (i.e., into the region of the first trigeminal branch). Furthermore, we were interested in differences between female and male subjects since pain perception also depends on sex and since the epidemiology of TACs shows remarkable differences between sexes.

## Methods

We enrolled healthy subjects (volunteers recruited from students and staff at the University of Münster) without any history of a primary headache disorder, only infrequent episodic tension-type headache was allowed. Even in first degree relatives, no migraine or TAC history was allowed. Subjects had to be without any medication (except hormonal contraception) including painkillers in the week before. The subjects were examined once, female subjects were examined outside their period of menstruation. They received an injection of capsaicin (0.05 ml; 0.01%) subcutaneously into the right forehead. The appearance of ipsilateral autonomic symptoms was carefully observed and documented. We registered the occurrence and the duration of:

- miosis.
- ptosis.
- lacrimation.
- conjunctival injection.
- nasal congestion.

The symptoms were all registered ipsilateral to the pain. Subjects rated their pain 1, 2, 5, and 10 min after injection of capsaicin on a visual numerical scale from 0 (no pain) to 10 (maximal pain). We also calculated a score for the autonomic activation by giving 1 point if a single symptom lasted 6 min or less and by giving 2 points if a single symptom lasted more than 6 min.

Results are presented as arithmetic mean +/- standard deviation and as percentage. For comparing the pain intensity and the autonomic score between sex, the nonparametric Mann-Whitney-U-test was used. For analysis of correlation between the pain intensity and the autonomic score, the Spearman-rank-coefficient was used. All statistical analyses were performed using SPSS version 15.0 (SPSS, Chicago, IL, USA). The level of significance was set at *p* < 0.05. Clinical trial number: not applicable.

## Results

We enrolled 60 healthy subjects (30 male and 30 female) with a mean age of 28 +/- 5 years (female: 26 +/- 4; male: 29 +/- 6) and a mean height of 176 +/- 10 cm (female: 171 +/- 12 cm; male: 182 +/-_9 cm) and a mean body weight of 71 +/- 13 kg (female: 63 +/- 15 kg; male: 78 +/- 11 kg). The pain intensity was rated between 6 and 10 by all subjects within one minute after capsaicin injection. The pain distribution was periorbital and frontal on the side of injection. The mean pain intensity for all measures is presented in Table 1 separately for female and male subjects. At all time points, female subjects rated the pain significantly higher than male subjects.

**Table 1:** Mean pain intensity 1, 2, 5, and 10 min after capsaicin injection into the right forehead presented as arithmetic mean and standard deviation. The pain was rated between 0 and 10 on a visual numeric scale. Comparison between female and male subjects by Mann-Whitney-U-test

	Female (*n*=30)	Male (*n*=30)	Significance
1 min	9.2 1.1	8.5 1.2	*P*=0.012
2 min	8.4 1.3	7.5 1.5	0.009
5 min	5.6 1.9	4.0 1.9	0.001
10 min	3.3 1.4	2.4 1.4	0.015

All subjects except two developed ipsilateral autonomic symptoms. There were only two subjects with contralateral autonomic symptoms. The most often reported autonomic symptom was lacrimation (84%); the least often reported autonomic symptoms were ptosis and nasal congestion (27%). The frequency and duration of the autonomic symptoms are presented in Table 2. For all autonomic symptoms except ptosis, the duration of the symptom was significantly longer in female than in male subjects. Also, the frequency of autonomic symptoms was higher in female subjects for all symptoms. Subsequently, the autonomic score as calculated in this study was significantly higher for female subjects.

**Table 2:** Frequency (as percentage) and duration (in minutes) of autonomic symptoms. The significance refers to the difference in duration

	Female	Male	Significance
Miosis			
Frequency	60%	27%	
Duration in minutes	7 +/- 3	4 +/- 2	*P*=0.003
Ptosis			
Frequency	43%	10%	
Duration	7 +/- 6	9 +/- 7	ns
Lacrimation			
Frequency	90%	77%	
Duration	9 +/- 4	5 +/- 3	*P*=0.005
Conjunctival injection			
Frequency	80%	20%	
Duration	7 +/- 5	3 +/- 1	*P*=0.044
Nasal congestion			
Frequency	40%	13%	ns
Duration in minutes	3 +/- 1	3 +/- 2	
Autonomic score	4.4 +/- 1.6	1.7 +/- 0.9	*P*<0.001

When correlating the autonomic score with the pain intensity after one minute, we received a Spearman-rank-coefficient of *r* = 0.425 (*p* < 0.001). Also, for the other time points of pain measuring, the correlation between the autonomic score and the pain intensity remained significant (data not shown). Even after adjusting for sex, the correlation between autonomic score and pain intensity remained significant although the correlation was much weaker. When analysing the sex groups separately, this correlation was only seen in female subjects (*r* = 0.477; *p* = 0.004) but not in male subjects (*r* = 0.031; ns).

## Discussion

In this experimental study, immediately after injection of capsaicin, strictly ipsilateral symptoms of autonomic activation occurred together with very severe pain in nearly all our healthy participants confirming previous findings in a small pilot study [7]. Since this was observed after activation of the first trigeminal branch, we speculate that there is an indirect functional connectivity between this nerve branch and the superior salivatory nucleus which is consistent with earlier observations showing also parasympathetic activation in the first trigeminal branch after capsaicin injection [8, 9]. However, this assumption is highly speculative since an anatomic connectivity has not been show to date. Autonomic symptoms in an experimental pain model suggest that severe pain in the first branch are sufficient to activate pathways involved in the autonomic system without a superimposed primary headache disorder. The data are consistent with the notion that pain triggers non-specific changes in vessel diameter not only in migraine and cluster headache but also after capsaicin injection into the forehead [8].

Another finding of this study is that frequency and duration of autonomic symptoms increased with the intensity of the pain caused by capsaicin in the first minutes of the observation period. Interestingly, in previous studies this increase of duration and frequency was only observed after injecting capsaicin into the region of the first trigeminal branch but not into the region of the third trigeminal branch (i.e., mandibular region) [7]; [9]. Therefore, we conclude that autonomic activation by pain occurs (almost) exclusively by triggering nociception in the first trigeminal branch.

The frequency of the different autonomic symptoms in our subjects is similar to the distribution seen in cluster headache with lacrimation and conjunctival injection showing the highest frequency [11]. However, autonomic symptoms seem also to depend on the region in the world and cannot be compared one to one between different genetic populations [12].

Another major finding of our study is that the female subjects had higher pain scores and showed more frequently autonomic symptoms with longer duration resulting in an increased autonomic score as compared to the male subjects. Higher pain rating on different pain scales after an external pain stimulation has been reported for female subjects in several studies [13]; [14]. However, we are not aware of studies reporting higher frequency and longer duration of autonomic symptoms in female subjects as compared to male subjects, neither in experimental pain nor in TACs except a higher rate of ptosis in female cluster headache patients [15]. The higher level of autonomic activation is only in part due to the higher pain ratings by female subjects. We cannot conclude on the mechanism why female subjects show higher pain rating and higher autonomic activation after the same highly painful stimulation in the first trigeminal branch than male subjects. Therefore, further investigations on experimental trigeminal pain considering the hormonal status, neuropeptide release, and other mechanisms are warranted.

Autonomic symptoms can also be induced by the pain of other primary headache disorders or facial pain disorders such as migraine [5]; [16]; [17]) and trigeminal neuralgia [6] or even in secondary headache disorders [18]. In epidemiological studies, between 13% and 37% of all migraine patients showed unilateral autonomic symptoms [5]; [16]; [19]; interestingly, these patients had more severe pain and more strictly unilateral pain than the other migraine patients. As in our study, the most prominent autonomic symptom was lacrimation, the least frequent symptoms were ptosis and nasal congestion. Those data and our data suggest that the autonomic activation is not specific for the group of TACs but rather an unspecific result of severe pain in the first trigeminal branch. We could recently show that also the release of CGRP is not specific to the group of TACs or to migraine since also healthy subjects release CGRP after capsaicin injection into the skin of the forehead [9]. We conclude that this mechanism is most likely caused by activation of the sphenopalatine ganglion reflex which can be regarded as an unspecific reflex to pain.

The question remains whether the autonomic activation seen in TACs is also an unspecific reaction of the severe pain or whether there are specific mechanisms in these headache disorders also leading to autonomic activation. However, autonomic symptoms without pain in patients with cluster headache [20–23] and in patients with chronic paroxysmal hemicrania [23]; [24] have been reported. This phenomenon illustrates that the nociceptive input is not a conditio sine qua non for the autonomic activation in these headache disorders and that another pathway must exist. In this context, it is remarkable that a patient with typical chronic cluster who underwent a trigeminal nerve sensory root section (denervating the ipsilateral cranial vessels and dura mater) neither had a stop of his attacks nor had any changes in the cranial autonomic symptoms affected by this procedure [21]. This suggests that the autonomic pathway resulting in lacrimation, rhinorrhoea, and conjunctival injection can be activated by nociception without the trigemino-facial (parasympathetic) reflex connection. It is also noteworthy that in our participants the autonomic symptoms outlasted the immediate severe pain sensation, which is usually severe for about 2 min following the injection.

We conclude that even short nociceptive activation in the first trigeminal branch is a trigger for autonomic activation in healthy subjects. It might be that in idiopathic headache disorders the trigeminal nociceptive activation also initiates α-CGRP release but that this release again increases pain intensity leading to a vicious cycle with autonomic activation. It should be noted in this context that the α-CGRP level increases by about 100% in migraine or even more in cluster headache as compared to baseline whereas in healthy subjects the increase is only about 40% [10]. Trigeminal nociception seems to activate a trigeminal-autonomic reflex regardless of whether this nociceptive activation develops in the course of a primary or secondary headache symptom, or experimentally induced like in our study.

There are some limitations to be discussed with respect to this study. First, we did not investigate the second and third branch, since these nerves are normally not involved in idiopathic headache disorders. However, there are patients with idiopathic headache disorders such as migraine (so-called facial migraine) and TACs in whom the second trigeminal branch is even more affected than the first trigeminal branch. A previous study showed that capsaicin injection in the region of the third trigeminal branch did not induce autonomic activation [10]. Second, the subject sample was quite homogenous with respect to age and body mass index. It is difficult to find volunteers for such painful studies who never had migraine or a TAC themselves or in their family; therefore, we mainly recruited medical students as volunteers. Third, we studied all female subjects regardless of their menstrual cycle. It has been shown that the menstrual cycle can have impact on pain perception also in the trigeminal region [25]. Although we did not measure during the period of menstruation, we cannot exclude that the menstrual cycle could have had an influence on the pain measures in our female subjects. Also, psychosocial factors might have caused the difference between female and male subjects, at least regarding pain ratings. However, it is unlikely that the autonomic symptoms themselves have been influenced by psychosocial factors. A link between sex and the severity of autonomic symptoms might be explained by the higher pain ratings and, thus, by an indirect influence of psychosocial factors. Last, we used a fixed amount of capsaicin to induce autonomic symptoms, which was also done by previous studies [8, 9]. It might, however, be that body mass index and sex influence the metabolism of capsaicin. It might be, for example, that capsaicin is metabolized in the soft tissue differently between female and male subjects; to our knowledge, this has not yet been studied. Future studies should analyse also the influence of different doses of capsaicin.

## Conclusion

Capsaicin injected in the first trigeminal branch region of healthy subjects induced ipsilateral autonomic symptoms representing parasympathetic activation. Frequency and duration of the autonomic symptoms showed a positive correlation with the pain intensity after capsaicin injection. Autonomic activation was much more pronounced in female subjects than in male subjects due to both a higher pain intensity experienced by female subjects and an unknown independent mechanism.

## Data Availability

The data that support the findings of this study are not openly available due to reasons of sensitivity and are available from the corresponding author upon reasonable request.
